# Effect of NBCe1 deletion on renal citrate and 2‐oxoglutarate handling

**DOI:** 10.14814/phy2.12778

**Published:** 2016-04-25

**Authors:** Gunars Osis, Mary E. Handlogten, Hyun‐Wook Lee, Kathleen S. Hering‐Smith, Weitao Huang, Michael F. Romero, Jill W. Verlander, I. David Weiner

**Affiliations:** ^1^ Division of Nephrology, Hypertension and Renal TransplantationUniversity of Florida College of MedicineGainesvilleFlorida; ^2^ Renal DivisionTulane University College of MedicineNew OrleansLouisiana; ^3^ Department of Physiology & Biomedical Engineering and Nephrology & HypertensionMayo Clinic College Of MedicineRochesterMinnesota; ^4^ Nephrology and Hypertension SectionNorth Florida/South Georgia Veterans Health SystemGainesvilleFlorida

**Keywords:** 2‐oxoglutarate, acidosis, citrate, NaDC‐1, NBCe1, proximal tubule

## Abstract

The bicarbonate transporter, NBCe1 (SLC4A4), is necessary for at least two components of the proximal tubule contribution to acid‐base homeostasis, filtered bicarbonate reabsorption, and ammonia metabolism. This study's purpose was to determine NBCe1's role in a third component of acid‐base homeostasis, organic anion metabolism, by studying mice with NBCe1 deletion. Because NBCe1 deletion causes metabolic acidosis, we also examined acid‐loaded wild‐type adult mice to determine if the effects of NBCe1 deletion were specific to NBCe1 deletion or were a non‐specific effect of the associated metabolic acidosis. Both NBCe1 KO and acid‐loading decreased citrate excretion, but in contrast to metabolic acidosis alone, NBCe1 KO decreased expression of the apical citrate transporter, NaDC‐1. Thus, NBCe1 expression is necessary for normal NaDC‐1 expression, and NBCe1 deletion induces a novel citrate reabsorptive pathway. Second, NBCe1 KO increased 2‐oxoglutarate excretion. This could not be attributed to the metabolic acidosis as experimental acidosis decreased excretion. Increased 2‐oxoglutarate excretion could not be explained by changes in plasma 2‐oxoglutarate levels, the glutaminase I or the glutaminase II generation pathways, 2‐oxoglutarate metabolism, its putative apical 2‐oxoglutarate transporter, OAT10, or its basolateral transporter, NaDC‐3. In summary: (1) NBCe1 is necessary for normal proximal tubule NaDC‐1 expression; (2) NBCe1 deletion results in stimulation of a novel citrate reabsorptive pathway; and (3) NBCe1 is necessary for normal 2‐oxoglutarate metabolism through mechanisms independent of expression of known transport and metabolic pathways.

## Introduction

Renal acid‐base homeostasis involves multiple interdependent mechanisms. These include filtered bicarbonate reabsorption, intrarenal ammonia metabolism and transport, and excretion of organic anions, such as citrate and 2‐oxoglutarate (Palmer and Alpern 2010; Weiner and Verlander 2012; Hamm et al. 2013; Weiner et al. 2013). Each has a critical role in multiple components of renal physiology. Filtered bicarbonate reabsorption is necessary for preventing renal tubular acidosis, which can cause, among other effects, severe failure to thrive and growth retardation in children, and metabolic acidosis with osteoporosis in adults (Rocher and Tannen 1986; Soriano 2002). Ammonia metabolism is the primary component of basal net acid excretion and variations in ammonia excretion are the quantitatively greatest component of the response to most acid‐base disturbances (Weiner and Hamm 2007; Weiner and Verlander 2013). Organic anion excretion is important because it enables alkali excretion without increasing urine pH, because it can regulate urinary acidification and sodium transport, and, for citrate, because it contributes to prevention of calcium nephrolithiasis (Hamm 1990; Packer et al. 1995; Cheema‐Dhadli et al. 2002; Unwin et al. 2004; Peti‐Peterdi 2013).

The proximal tubule has a central role in the renal regulation of each of these components of acid‐base homeostasis. The majority of filtered bicarbonate is reabsorbed in the proximal tubule (Boron 2006; Skelton et al. 2010; Zhuo and Li 2013). Renal ammonia metabolism requires intrarenal ammonia generation, and the proximal tubule is the primary site of renal ammoniagenesis (Wright and Knepper 1990; Weiner and Verlander 2013; Curthoys and Moe 2014). Organic anion excretion is regulated predominantly, if not exclusively, through regulated reabsorption of filtered organic anions in the proximal tubule (Hamm 1990; Unwin et al. 2004). Thus, the proximal tubule mediates an essential role in acid‐base homeostasis.

The proximal tubule sodium‐coupled, electrogenic bicarbonate transporter, isoform 1, NBCe1 (SLC4A4), has a critical role in multiple aspects of the proximal tubule contribution to acid‐base homeostasis. Filtered bicarbonate reabsorption requires efficient bicarbonate movement across the basolateral plasma membrane into the peritubular space, a process mediated primarily by the electrogenic sodium‐coupled bicarbonate transporter, isoform 1 (NBCe1, SLC4A4) (Boron 2006; Kurtz and Zhu 2013; Zhuo and Li 2013). Inhibiting NBCe1 blocks bicarbonate reabsorption, and genetic deletion or defects in NBCe1 leads to the clinical scenario known as proximal renal tubular acidosis (RTA) (Igarashi et al. 1999; Kurtz and Zhu 2013, 2013). NBCe1 also appears to have a critical role in ammonia metabolism. Our recent studies showed that genetic deletion of NBCe1 results in altered renal ammonia metabolism involving dysregulated expression of multiple proteins involved in proximal tubule ammonia metabolism, including phosphate‐dependent glutaminase, phosphoenolpyruvate carboxykinase, and glutamine synthetase (Handlogten et al. 2015).

The purpose of the current studies was to determine NBCe1's role in renal organic anion transport. We examined mice with genetic deletion of NBCe1 that have been shown previously to develop proximal RTA (Gawenis et al. 2007) and to have significant abnormalities in renal ammonia metabolism (Handlogten et al. 2015). Because NBCe1 deletion results in early death (Gawenis et al. 2007), we studied mice at day 8 ± 1 of age. First, we determined the effect of NBCe1 deletion on urinary citrate and 2‐oxoglutarate excretion. Because NBCe1 deletion causes metabolic acidosis (Gawenis et al. 2007; Handlogten et al. 2015), which also alters renal citrate and 2‐oxoglutarate metabolism (Hamm 1990; Unwin et al. 2004; Moe and Preisig 2006), we contrasted the effects of NBCe1 deletion with those of experimentally induced metabolic acidosis in adult WT mice. We then determined the effect of NBCe1 deletion on expression of the primary renal citrate and 2‐oxoglutarate transporters and on the primary enzymes involved in 2‐oxoglutarate metabolism. Finally, because elevated 2‐oxoglutarate excretion normally is associated with urine alkalinization (Tokonami et al. 2013), which is not present in mice with NBCe1 deletion (Handlogten et al. 2015), we examined the effect of NBCe1 deletion on the 2‐oxoglutarate receptor, Oxgr1 (He et al. 2004a), and on the putative target of Oxgr1 activation, pendrin (Tokonami et al. 2013).

## Methods

### Animals

Mice with NBCe1 deletion have been described previously (Gawenis et al. 2007; Handlogten et al. 2015). Heterozygous mice were bred in the University of Florida Cancer and Genetics Transgenic Animal Facility by trained personnel. We genotyped all mice using DNA obtained from tail‐clip specimens using standard techniques (Gawenis et al. 2007; Bishop et al. 2013; Lee et al. 2013b, 2014, 2015). Mice with homozygous NBCe1 deletion and their wild‐type littermates were used for these studies. Mice were studied at day 8 ± 1 as described previously (Handlogten et al. 2015). Mice with NaDC‐1 gene deletion have been described previously (Ho et al. 2007) and were housed and bred in the Tulane University Animal Care Facility. All animal studies were approved by the Institutional Animal Care and Use Committees at University of Florida College of Medicine and the North Florida/South Georgia Veterans Health System or at Tulane University.

### Metabolic acidosis

Metabolic acidosis was generated in adult C57Bl/6 mice using methods we have described in detail previously (Lee et al. 2009, 2013b, 2014; Bishop et al. 2013). Adult animals were placed into metabolic cages (Tecniplast diuresis metabolic cage, Fisher Scientific). Urine was collected under mineral oil, and daily urine volume and pH were recorded. Urine samples were stored at −20°C until analyzed further. Animals were allowed to acclimate for 2 days while receiving the control diet and then were allocated to either the acid or control diet. Acid diet was generated by adding 0.4 mol/L HCl to powdered standard rodent chow in a ratio of 1 mL/g chow as described previously (Lee et al. 2009, 2010, 2014; Bishop et al. 2010). The control diet was identical, except deionized water substituted for HCl. After 7 days, mice were anesthetized with inhalant isoflurane and the kidneys were flushed free of visible blood by in vivo cardiac perfusion with PBS (pH 7.4). The right kidney was rapidly removed, the renal cortex dissected free and flash‐frozen in liquid nitrogen or transferred to RNAlater (Life Technologies, Inc.) and placed at 4°C for 24 h. Samples were then stored at −70°C for protein isolation or total RNA extraction at a later time. The left kidney was preserved by perfusion with 2% paraformaldehyde‐lysine‐periodate (PLP) followed by immersion in PLP for 24 h at 4°C.

### Mouse pup tissue collection

Mouse pups were anesthetized with inhalant isoflurane. Kidneys were excised and either transferred to PLP fixative and stored at 4°C for 24–48 h before processing for immunohistochemistry, or processed as described above for protein and mRNA isolation.

### Urinary studies

Urine was collected from mouse pups by bladder puncture following euthanasia or by immediate aspiration following spontaneous voiding during the anesthesia process and was frozen for subsequent analysis. Urine creatinine was measured by the modified version of the Jaffe reaction, in which creatinine was treated with an alkaline picrate solution to yield a colored complex, as described recently (Handlogten et al. 2015). Urinary 2‐oxoglutarate was measured using a 2‐oxoglutarate Colorimetric Assay Kit (MAK054, Sigma Aldrich) according to the manufacturer's directions. For the citrate assays we used a Citrate Colorimetric Assay Kit (BioVision, Cat #K655‐100).

### Antibodies

Antibodies to NaDC‐1, glutamine transaminase K (GTK), and *ω*‐amidase were obtained from Proteintech Group, Inc. (Rosemont, IL). Antibodies to pendrin were graciously supplied by Susan M. Wall, M.D. (Emory University) (Knauf et al. 2001).

### Protein preparation

Snap frozen tissues were homogenized in T‐PER (Tissue Protein Extraction Reagent, Pierce Biotechnology, Rockford, IL) using microtube pestles (USA Scientific, Ocala, FL), and protein was extracted according to the manufacturer's recommended procedures. An aliquot was used for total protein quantification using a BCA assay, and the remainder was stored frozen at −70°C until used.

### Immunoblotting procedure

Immunoblotting was performed as we have described previously (Kim et al. 2007; Bishop et al. 2010; Lee et al. 2010). Briefly, 20 micrograms of renal protein was electrophoresed on 10% PAGE ReadyGel (Bio‐Rad, Hercules, CA). Gels were then transferred electrophoretically to nitrocellulose membranes, blocked with 5 g/dL nonfat dry milk in Blotto buffer (50 mmol/L Tris, 150 mmol/L NaCl, 5 mmol/L Na_2_EDTA, and 0.05% Tween 20, pH 7.6), and incubated at 4°C overnight with primary antibody diluted in nonfat dry milk. Loading and transfer equivalence were assessed with Ponceau S staining. After washing, membranes were exposed to secondary antibody, goat anti‐rabbit IgG (Millipore, Billerica, MA), conjugated to horseradish peroxidase at a dilution of 1:5,000. Sites of antibody‐antigen reaction were visualized by using enhanced chemiluminescence (SuperSignal West Pico Substrate, Pierce) and a Kodak Image Station 440CF digital imaging system. In selected experiments, blots were stripped and expression of the house‐keeping protein, beta‐actin, was determined. Band density was quantified using Kodak 1D, version 5.0, software (Kodak Scientific Imaging, New Haven, CT). Band density was normalized such that mean density in wild‐type tissues was 100. The absence of saturation was confirmed by examining pixel intensity distribution in all immunoblots.

### Tissue preparation for immunolocalization

PLP‐preserved kidneys were embedded in polyester wax made using polyethylene glycol 400 distearate (Polysciences, Warrington, PA) with 10% 1‐hexadecanol, and 3‐*μ*m‐thick sections were cut and mounted on gelatin‐coated glass slides.

### Immunohistochemistry

Immunolocalization was accomplished using standard immunoperoxidase procedures in our laboratory (Bishop et al. 2013; Lee et al. 2013b, 2014; Verlander et al. 2013). Briefly, sections were dewaxed in ethanol, rehydrated, and then rinsed in PBS. For some experiments, antigen retrieval was accomplished by immersing slides in Trilogy^™^ (Cell Marque, Rocklin, CA) and heating to 88–96°C for 1 h. Endogenous peroxidase activity was blocked by incubating the sections in 3% H_2_O_2_ in distilled water for 45 min. The sections were blocked for 15 min with Serum‐Free Protein Block (Dako Cytomation) and were then incubated at 4°C overnight with primary antibody. The sections were washed in PBS and incubated for 30 min with polymer‐linked, peroxidase‐conjugated goat anti‐rabbit IgG (MACH2, Biocare Medical, Concord, CA), again washed with PBS, then exposed to diaminobenzidine (DAB) for 5 min. The sections were washed in distilled water, dehydrated in a graded series of ethanol and xylene, mounted, and observed by light microscopy. Comparisons of labeling were made only between sections from the same immunohistochemistry experiment.

Sections were examined on both a Nikon E600 microscope equipped with DIC optics and photographed using a DXM1200F digital camera and ACT‐1 software (Nikon) and on a Leica DM2000 microscope and photographed using a Leica DFC425 digital camera and Leica DFC Twain Software and LAS application suite (Leica Microsystems, Buffalo Grove, IL). Color adjustment and contrast enhancement was performed using Adobe Photoshop CS2 and CS5 software (Adobe Systems, San Jose, CA).

### mRNA extraction

Total RNA was extracted from kidney tissues using RNeasy Mini Kit (Qiagen) according to the manufacturer's instructions and stored in a −70°C freezer until used.

### Real‐time RT‐PCR

Real‐time RT‐PCR amplification was performed as described previously (Weiner et al. 2003; Handlogten et al. 2005; Lee et al. 2013a). Briefly, total RNA was reverse‐transcribed using SuperScript First Strand Synthesis System for RT‐PCR (Invitrogen, Carlsbad, CA) and random hexamer primers. Taqman primer‐probe sets for glutamate dehydrogenase, oxoglutarate dehydrogenase, dihydrolipoyl succinyltransferase, dihydrolipoyl dehydrogenase, OAT10, Oxgr1, and GAPDH were obtained from Life Technologies, Inc., Grand Island, NY. Real‐time RT‐PCR was performed on an ABI Prism GeneAmp 7500 Sequence Detection System. We used a two‐step cycle protocol including an initial 95°C denaturation step for 15 sec and then 60°C for 1 min, and then 40–50 cycles of alternating temperatures. GAPDH mRNA was amplified in all experiments as an internal control. All experiments included samples not treated with reverse transcription and samples to which RNA was not added as internal controls.

We calculated relative mRNA expression using the standard formula: $\text{Relative}\;{\text{mRNA}}_{x}=2^{-({\text{ct}}_{x}-{\text{ct}}_{\mathrm{GAPDH}})}$. All amplification curves were examined to confirm that fluorescence intensity increased in an exponential pattern during the amplification phase. We normalized each mRNA measurement to the mean for the appropriate control group. For studies examining the effect of NBCe1 deletion, we used the mean expression in wild‐type age‐matched littermates as the control group, and normalized this to 100%. For studies examining the effect of experimental metabolic acidosis, we used the mean expression in non‐acid‐loaded mice as the control group and normalized this to 100%.

### Statistics

Results are presented as means ± SE. Statistical analyses were performed using Student's two‐tailed t‐test or ANOVA. *P *<* *0.05 was taken as statistically significant; *n* refers to the numbers of animal studied.

## Results

### NBCe1 deletion and organic anion excretion

**Figure 1:**
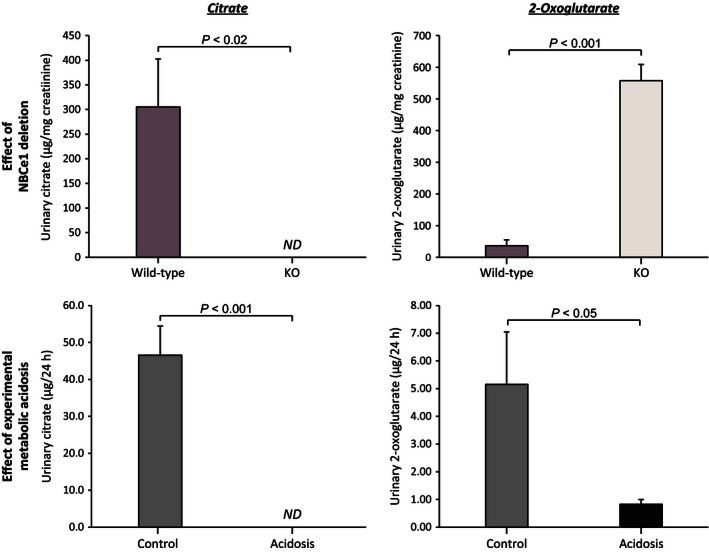
Effect of NBCe1 deletion and of experimental metabolic acidosis on urinary citrate and 2‐oxoglutarate excretion. *Top left panel* shows effects of NBCe1 deletion on citrate excretion. NBCe1 KO resulted in no detectable (ND) urinary citrate excretion. *Top right panel* shows effects of NBCe1 deletion on 2‐oxoglutarate excretion. Mice with NBCe1 deletion excreted significantly more 2‐oxoglutarate than did wild‐type littermates. *Bottom left panel* shows effects of experimental metabolic acidosis on urinary citrate excretion in adult wild‐type mice. Metabolic acidosis in adult wild‐type mice resulted in complete suppression of urinary citrate excretion. *Bottom right panel* shows effects of experimental metabolic acidosis on 2‐oxoglutarate excretion in adult wild‐type mice. Metabolic acidosis significantly decreased 2‐oxoglutarate excretion. *N* = 4 in each group in each experiment. Note that the units are different in the measurements performed in pup kidneys, *μ*g per mg creatinine, from those in the experimental metabolic acidosis studies, *μ*g per 24 h. ND, not detectable.

Our first studies examined the effect of NBCe1 deletion on urinary organic anion excretion. Figure 1 summarizes the results. NBCe1 deletion was associated with a significant decrease in citrate excretion (WT, 305 ± 97 *μ*g/mg creatinine; vs KO, 0.0 ± 0.0 *μ*g/mg creatinine; *n* = 5 in each group; *P* < 0.02). 2‐oxoglutarate excretion, in contrast, was significantly increased in mice with NBCe1 deletion as compared to wild‐type mice (WT, 36.5 ± 18.5 *μ*g/mg creatinine; vs. KO, 557.7 ± 51.2 *μ*g/mg creatinine; *n* = 5 in each group; *P* < 0.001).

Mice with NBCe1 deletion spontaneously develop metabolic acidosis (Gawenis et al. 2007; Handlogten et al. 2015), which alone can alter organic anion metabolism. In order to determine the independent effects of metabolic acidosis on organic anion excretion, we examined urine from adult wild‐type mice^1^ with experimentally induced metabolic acidosis. Figure 1 summarizes these results. In wild‐type mice, metabolic acidosis decreased both citrate (control, 46.6 ± 7.9 *μ*g per 24 h; vs. acidosis, 0.0 ± 0.0 *μ*g per 24 h; *n* = 5 in each group; *P* < 0.001) and 2‐oxoglutarate excretion (WT, 5.15 ± 1.90 *μ*g per 24 h; vs. acidosis, 0.83 ± 0.17 *μ*g per 24 h; *n* = 5 in each group; *P* < 0.05) significantly. These findings indicate that the effects of NBCe1 deletion on citrate excretion are similar to those observed with metabolic acidosis, but that the effects on 2‐oxoglutarate excretion are diametrically different.

### Effect of NBCe1 deletion on plasma citrate and 2‐oxoglutarate

Plasma levels of citrate averaged 82.5 ± 7.1 and 87.2 ± 11.8 ng/*μ*L in wild‐type and NBCe1 knockout mice, respectively. NBCe1 deletion did not significantly alter plasma citrate levels (*n* = 3 and 4, respectively, *P* = NS). Similarly, plasma levels of 2‐oxoglutarate did not differ significantly between as a result of NBCe1 deletion (wild‐type, 11.8 ± 4.5; and KO, 4.5 ± 0.7 μmol/L, *N* = 5 and 8, respectively, *P* = NS).

### NBCe1 deletion and NaDC‐1 expression

**Figure 2:**
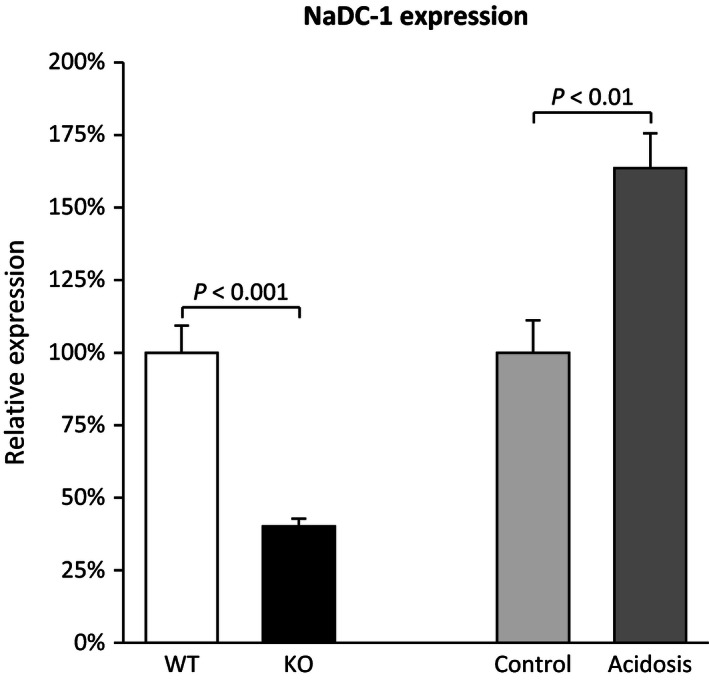
Effect of NBCe1 deletion on NaDC‐1 expression. *Left side* of figure shows that NBCe1 deletion decreased NaDC‐1 mRNA expression significantly. *Right side* shows that experimental metabolic acidosis, examined in adult wild‐type mice, in contrast increased NaDC‐1 mRNA expression significantly. Because NBCe1 deletion causes spontaneous metabolic acidosis (Gawenis et al. 2007; Handlogten et al. 2015), these results indicate that NBCe1 expression is necessary for the normal regulation of NaDC‐1 expression. NaDC‐1 mRNA expression was determined using Taqman real‐time RT‐PCR, and normalized to GAPDH expression. *N* = 4 in all samples. Results are normalized such that wild‐type expression, for pups, and control diet expression, for adult mice, is 100.0%. Because this approach is used in all experiments quantifying mRNA expression, this information is not repeated in subsequent figure legends.

**Figure 3:**
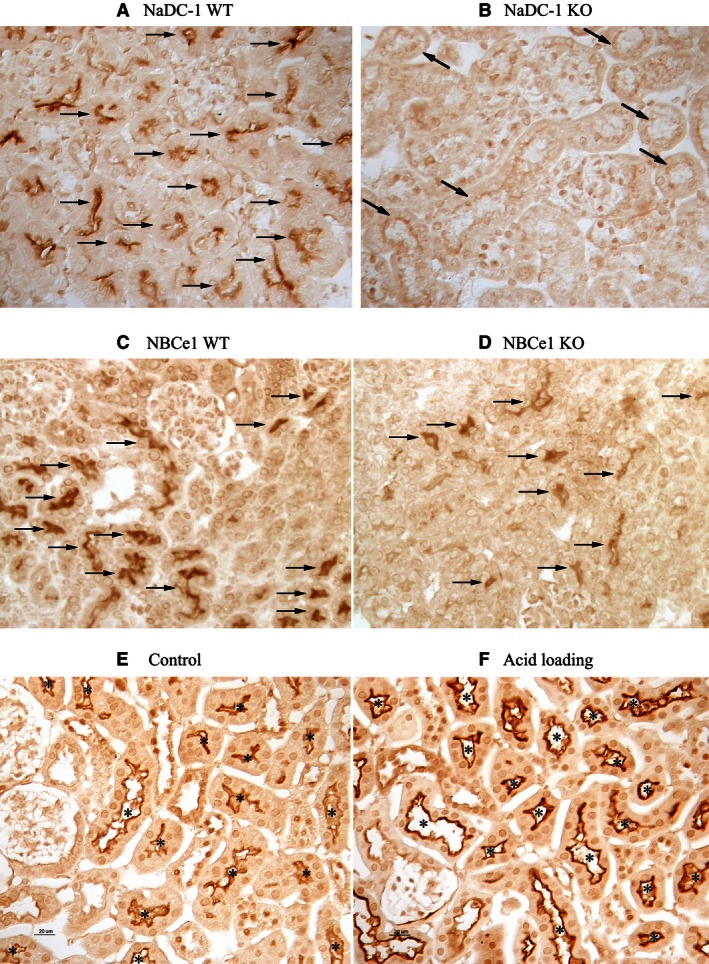
NaDC‐1 immunolabel in wild‐type and NBCe1 knock‐out pup kidneys. *Top panels* show characterization of NaDC‐1 antibody used in these studies. *Panel A* shows that apical immunolabel is present in proximal tubule segments (arrows) of wild‐type C57Bl/6 mice. *Panel B* shows that no detectable immunolabel is present in proximal tubule segments (identified by apical brush border) (black arrows) of mice with NaDC‐1 deletion. Results are representative of findings in four wild‐type and four NaDC‐1 KO kidneys. In *middle panels*,* Panel C* shows NaDC‐1 immunolabel in the cortex of wild‐type (WT) pups and *Panel D* shows NaDC‐1 immunolabel in the cortex of NBCe1 knockout (KO) kidney. Apical NaDC‐1 immunolabel intensity is decreased in proximal tubule cells (arrows) of NBCe1 KO as compared to wild‐type mice. In the *bottom panels*,* Panel E* shows NaDC‐1 immunolabel in the cortex of control mice and *Panel F* shows NaDC‐1 immunolabel in the cortex of acid‐loaded mice. Apical NaDC‐1 immunolabel is present in renal proximal tubule cells (“*”) and expression is increased in response to acid‐loading. Results are representative of findings in four mice in each condition.

Luminal citrate reabsorption via NaDC‐1 is believed generally to be the primary mechanism regulating renal citrate excretion (Pajor 1999, 2014; Unwin et al. 2004). Thus, we determined the effect of NBCe1 deletion on NaDC‐1 expression. Mice with NBCe1 deletion had significantly lower NaDC‐1 mRNA expression than did wild‐type littermates (WT, 100 ± 9%; KO, 40 ± 4%, *n* = 4 in both groups; *P* < 0.001, Fig. 2). Immunohistochemistry showed that both wild‐type and NBCe1 KO pups exhibited apical NaDC‐1 immunolabel, but that NBCe1 deletion substantially decreased immunolabel intensity (Fig. 3).

The effect of NBCe1 deletion on NaDC‐1 expression cannot be explained by the associated metabolic acidosis. In wild‐type mice, acid‐loading increased NaDC‐1 mRNA expression (control, 100 ± 11%; acidosis, 164 ± 12%; *n* = 4 in both groups; *P* < 0.01) and it increased proximal tubule apical NaDC‐1 immunolabel (Fig. 3).

These studies show both NBCe1 KO and experimental metabolic acidosis decrease urinary citrate excretion. However, experimental acidosis increases NaDC‐1 mRNA and protein expression, whereas NBCe1 KO decreases NaDC‐1 expression.

### NBCe1 deletion and 2‐oxoglutarate generation pathways

Because 2‐oxoglutarate excretion was markedly increased in NBCe1 null mice in contrast to mice with metabolic acidosis alone, we examined expression of key steps in 2‐oxoglutarate generation. The two major pathways for 2‐oxoglutarate generation involve the glutaminase I and the glutaminase II pathways. The glutaminase I pathway involves phosphate‐dependent glutaminase (PDG) and glutamate dehydrogenase, whereas the glutaminase II pathway involves glutamine transaminase K (GTK) and *ω*‐amidase. Our recent studies show that NBCe1 deletion decreases PDG expression (Handlogten et al. 2015), which cannot explain the increased 2‐oxoglutarate excretion, so this pathway was not examined further.

**Figure 4:**
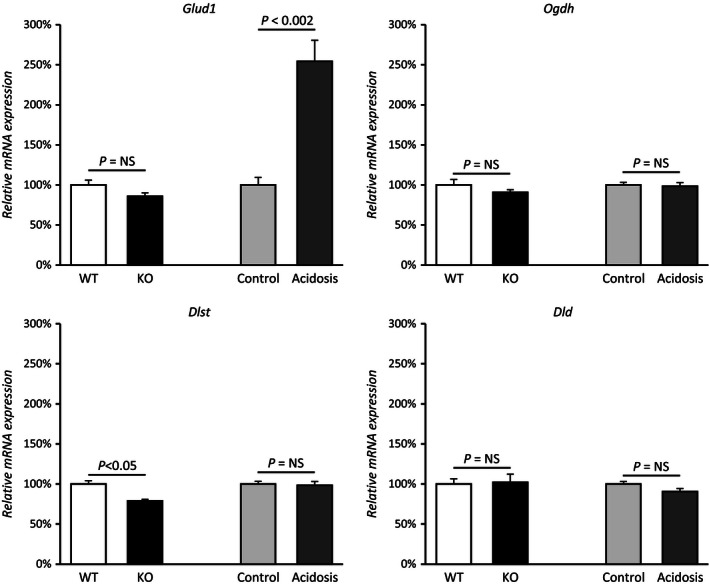
NBCe1 deletion and 2‐oxoglutarate metabolism. Effects of NBCe1 deletion on expression of key enzymes involved in 2‐oxoglutarate generation (Glud1) and metabolism (Ogdh, Dlst and Dld). Because NBCe1 causes spontaneous metabolic acidosis, adult wild‐type mice with experimentally induced metabolic acidosis were also examined to allow separate assessment of effects of NBCe1 deletion and metabolic acidosis. NBCe1 KO did not alter Glud1 expression significantly, whereas experimental metabolic acidosis increased it significantly. Because NBCe1 KO mice have metabolic acidosis, this indicates that NBCe1 expression is necessary for normal Glud1 response to metabolic acidosis. NBCe1 KO decreased Dlst expression slightly, but significantly. There were no effects of either NBCe1 KO or experimental metabolic acidosis on the other components of the 2‐oxoglutarate dehydrogenase complex, Ogdh or Dld. *N* = 4 in all samples.

Glutamate dehydrogenase (Glud1) catalyzes the oxidative deamination of glutamate to 2‐oxoglutarate and NH_4_^+^, enabling a role in the cellular production of both 2‐oxoglutarate and ammonia. As shown in Figure 4, NBCe1 deletion did not alter Glud1 expression significantly (WT, 100 ± 6%; KO, 86 ± 4%; *n* = 4 in each group, *P* = NS). These effects of NBCe1 deletion on Glud1 expression were very different than those from experimental metabolic acidosis. In kidneys from acid‐loaded mice, Glud1 expression increased significantly (control, 100 ± 9%; acidosis, 254 ± 26%; *n* = 4 in each group, *P* < 0.01, Fig. 4). Increased Glud1 expression with experimental metabolic acidosis is consistent with previous reports (Lombardo et al. 1981; Bogusky and Dietrich 1989; Mu and Welbourne 1996) and likely contributes to the increased ammonia generation that occurs during metabolic acidosis. The lack of Glud1 increase in the spontaneously acidotic NBCe1 KO mice is consistent with the generalized disturbance in proximal tubule ammonia metabolism reported recently (Handlogten et al. 2015).

**Figure 5:**
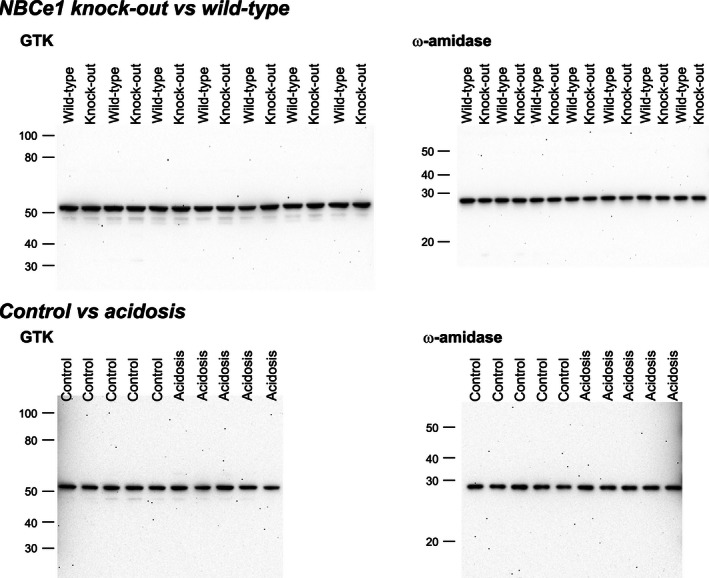
Effect of NBCe1 deletion on glutaminase II pathway. *Top left panel* shows effect of NBCe1 deletion on glutamine transaminase K (GTK) expression and *top right panel* shows effect on *ω*‐amidase expression using immunoblot analysis. NBCe1 deletion does not alter expression of either protein significantly. *Bottom left panel* shows effect of experimental metabolic acidosis on GTK expression and *bottom right panel* shows the effect of experimental metabolic acidosis on *ω*‐amidase expression. There was no significant effect of experimental metabolic acidosis on expression of either GTK or *ω*‐amidase.

A second mechanism of 2‐oxoglutarate generation involves the glutaminase II pathway, in which glutamine transaminase K (GTK) and *ω*‐amidase act in series. NBCe1 deletion did not alter expression of either GTK (WT, 100 ± 5%; KO, 104 ± 1%, *P* = NS, *n* = 7 in each group) or *ω*‐amidase (WT, 100 ± 6%; KO, 90 ± 5%; *P* = NS; *n* = 7 in each group) (Fig. 5). Thus, changes in 2‐oxoglutarate excretion with NBCe1 deletion do not appear to result from changes in the expression of proteins involved in the glutaminase II pathway.

Because NBCe1 deletion induced metabolic acidosis, and metabolic acidosis alters 2‐oxoglutarate excretion in the opposite direction as NBCe1 deletion, we could not exclude the possibility that NBCe1 deletion and metabolic acidosis have independent and counterbalancing effects on glutaminase II pathway proteins. To test this possibility, we examined tissues from adult wild‐type mice with experimentally induced metabolic acidosis (Fig. 5). There was no significant change in the expression of either GTK (Control, 100 ± 7%; Acidosis, 93 ± 10%; *P* = NS, *n* = 5 in each group) or *ω*‐amidase (Control, 100 ± 8%; Acidosis, 99 ± 4%, *P* = NS, *n* = 5 in each group). Thus, changes in urinary 2‐oxoglutarate appear unrelated to changes in expression of either of the proteins involved in the glutaminase II pathway, GTK or *ω*‐amidase.

### NBCe1 deletion and 2‐oxoglutarate metabolic pathway

The next step in cellular 2‐oxoglutarate metabolism we examined involves the 2‐oxoglutarate dehydrogenase complex, which consists of three separate components, oxoglutarate dehydrogenase (Ogdh), dihydrolipoyl succinyltransferase (Dlst), and dihydrolipoyl dehydrogenase (Dld). NBCe1 deletion did not significantly alter expression of either Ogdh (WT, 100 ± 7%; KO, 91 ± 3%; *n* = 4 in each group, *P* = NS) or Dld (WT, 100 ± 6%; KO, 102 ± 10%; *n* = 4 in each group, *P* = NS), but was associated with a small decrease in Dlst expression (WT, 100 ± 4%; KO, 79 ± 2%; *n* = 4 in each group, *P* < 0.05). Figure 4 summarizes these results.

Experimental metabolic acidosis had a similar lack of effect on components of the 2‐oxoglutarate dehydrogenase complex. Specifically, there was no significant change in Ogdh (control, 100 ± 7%; acidosis, 98 ± 3%; *n* = 4 in each group, *P *= NS), Dlst (control, 100 ± 4%; acidosis, 98 ± 2%; *n* = 4 in each group, *P* = NS), or Dld expression (control, 100 ± 6%; acidosis, 91 ± 10%; *n* = 4 in each group, *P *= NS, Fig. 4). Moreover, the effects of NBCe1 KO and experimental metabolic acidosis on Dlst expression did not differ significantly (*P* = NS by ANOVA). Thus, variations in 2‐oxoglutarate dehydrogenase complex expression are unlikely to be a major mechanism of the altered 2‐oxoglutarate excretion with either NBCe1 deletion or with metabolic acidosis alone.

### NBCe1 deletion and 2‐oxoglutarate transport mechanisms

**Figure 6:**
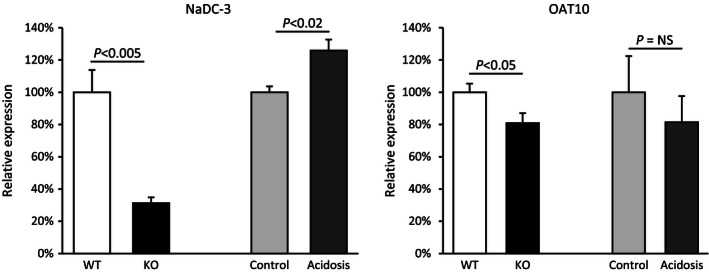
Effect of NBCe1 deletion on 2‐oxoglutarate transport mechanisms. *Left panel* shows expression of the basolateral organic anion transporter, NaDC‐3. NBCe1 KO decreased NaDC‐3 expression significantly. Experimental metabolic acidosis, in contrast, increased NaDC‐3 expression significantly. *Right panel* shows expression of the putative apical 2‐oxoglutarate transporter, OAT10. NBCe1 KO decreased OAT10 expression slightly, but significantly. Experimental metabolic acidosis induced a quantitatively similar, but not statistically significant, decrease. *N* = 4 in all samples.

The primary mechanism of proximal tubule basolateral 2‐oxoglutarate uptake involves the sodium‐coupled dicarboxylate transporter, NaDC‐3 (Kekuda et al. 1999; Burckhardt et al. 2005; Pajor 2014). NaDC‐3 expression in NBCe1 KO mice was decreased significantly compared to WT littermates (WT, 100 ± 14%; KO, 31 ± 4%, *n* = 4 in both groups, *P* < 0.005, Fig. 6). However, decreased NaDC‐3 expression, because this is a basolateral 2‐oxoglutarate uptake mechanism, is unlikely to explain the increase in 2‐oxoglutarate excretion in NBCe1 KO mice.

The specific mechanism of 2‐oxoglutarate secretion remains unclear, but recent studies suggest the organic anion transporter, OAT10, may mediate proximal tubule apical 2‐oxoglutarate secretion (Bahn et al. 2008; Grimm et al. 2015). As shown in Figure 6, NBCe1 KO was associated with a small, but statistically significant, decrease in OAT10 expression as compared to wild‐type littermates (WT, 100 ± 5%; KO, 81 ± 6%, *n* = 4 in all groups; *P* < 0.05). However, because OAT10 is thought to enable 2‐oxoglutarate secretion, decreased OAT10 expression is unlikely to explain the increased 2‐oxoglutarate excretion observed with NBCe1 deletion.

### Experimental metabolic acidosis and 2‐oxoglutarate transport mechanisms

As explained previously, because NBCe1 deletion caused metabolic acidosis, it was important to consider whether experimental metabolic acidosis alters either NaDC‐3 or OAT10 expression. The effects of experimental metabolic acidosis on 2‐oxoglutarate transport mechanisms differed from those of NBCe1 deletion. Acid‐loading increased NaDC‐3 expression significantly (Control, 100 ± 4%; acidosis, 126 ± 7%, *n* = 4 in both groups, *P* < 0.02), which contrasted to the decrease observed with NBCe1 KO (Fig. 6). However, increased NaDC‐3 expression, because this is a basolateral protein involved in cellular 2‐oxoglutarate uptake, is unlikely to mediate the decreased 2‐oxoglutarate excretion observed with experimental metabolic acidosis. Moreover, the differing effects of NBCe1 deletion and experimental metabolic acidosis on NaDC‐3 expression indicate that NBCe1 expression is necessary for normal NaDC‐3 expression.

As discussed above, OAT10 has been proposed to function as an apical 2‐oxoglutarate transport mechanism. Although the mean values for OAT10 expression in control versus experimental metabolic acidosis mirrored those for WT versus NBCe1 deletion, the difference was not statistically significant (Control, 100 ± 22%; Acidosis, 81 ± 16%, *n* = 4 in both groups, *P* = NS, Fig. 6). Thus, changes in OAT10 expression do not correlate with changes in 2‐oxoglutarate excretion, either in response to NBCe1 deletion or experimental metabolic acidosis.

### NBCe1 deletion and Oxgr1 expression

Luminal 2‐oxoglutarate increases in alkalosis and has been suggested to function as a paracrine signaling molecule activating the Oxgr1 receptor and stimulating bicarbonate secretion by pendrin‐positive collecting duct cells (Tokonami et al. 2013). If this occurred in the NBCe1 KO mice, the activation of pendrin‐mediated bicarbonate secretion would be expected to increase urine pH. However, mice with NBCe1 deletion actually have a urine pH that is significantly lower than in their wild‐type littermates (Handlogten et al. 2015).

**Figure 7:**
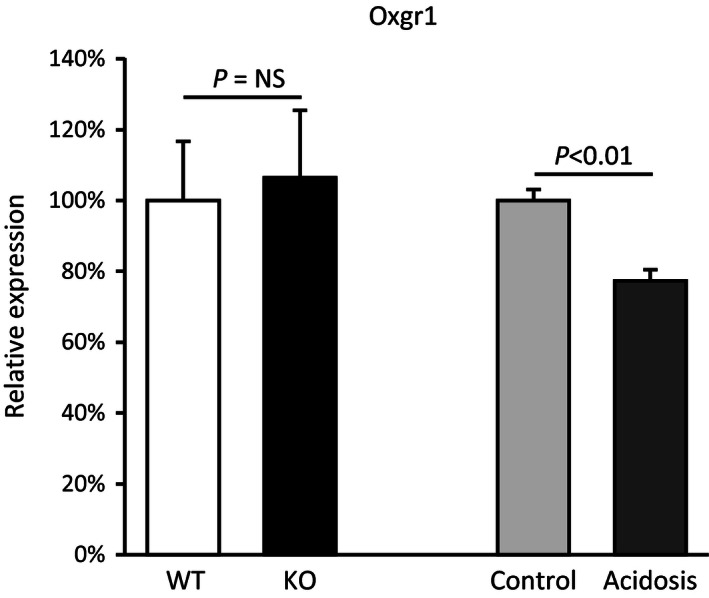
Effects of NBCe1 deletion and experimental metabolic acidosis on Oxgr1 mRNA expression. *Left bars* show effect of NBCe1 deletion on Oxgr1 expression. NBCe1 KO did not alter Oxgr1 expression significantly. *Right bars* show that experimental metabolic acidosis decreased Oxgr1 expression significantly. Results are normalized to WT expression, for pups, and to control diet, for adult mice. *N* = 4 in all samples.

In order to determine why urinary pH remained low despite high 2‐oxoglutarate excretion we first determined the effect of NBCe1 deletion on Oxgr1 expression. Figure 7 summarizes these findings. NBCe1 deletion did not alter Oxgr1 expression significantly (WT, 100 ± 17%; NBCe1 KO, 107 ± 17%; *n* = 4 in each group; *P* = NS). In contrast, experimental metabolic acidosis significantly decreased Oxgr1 expression (control, 100 ± 3%; vs. acid‐loading, 77 ± 3%; *n* = 4 in each group, *P* < 0.01). Thus, the absence of an increased urine pH in NBCe1 KO mice that have increased urinary 2‐oxoglutarate excretion cannot be ascribed to decreased expression of the 2‐oxoglutarate receptor, Oxgr1.

### NBCe1 deletion and pendrin expression

**Figure 8:**
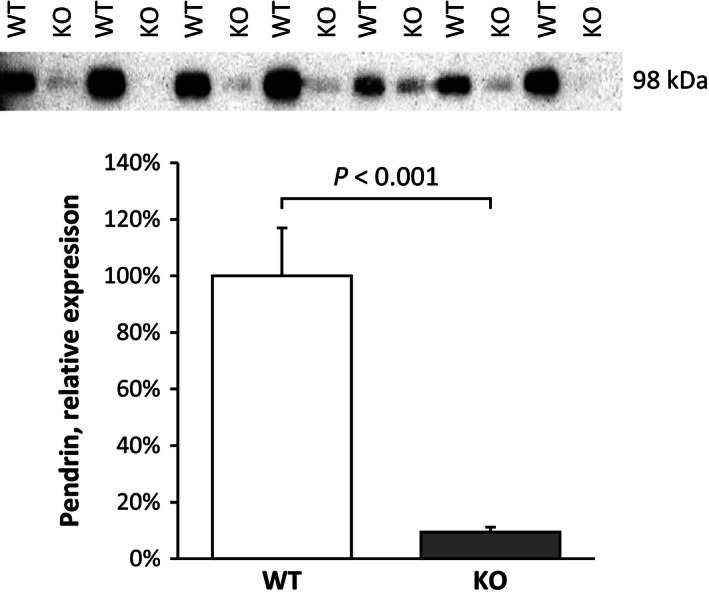
NBCe1 deletion alters pendrin protein expression. *Top panel* shows immunoblot assay for pendrin in wild‐type and NBCe1 KO pup kidneys. *Bottom panel* shows quantification of immunoblot analysis. NBCe1 deletion decreased pendrin protein expression significantly.

The 2‐oxoglutarate receptor, Oxgr1, is expressed in pendrin‐positive collecting duct cells. Luminal 2‐oxoglutarate is believed to increase renal alkali excretion by stimulating pendrin‐mediated bicarbonate secretion (Tokonami et al. 2013). In mice with NBCe1 deletion, pendrin protein expression, quantified by immunoblot analysis, was decreased significantly (Fig. 8). The absence of significant urine alkalinization in NBCe1 KO mice appears to be due, at least in part, to decreased expression of the 2‐oxoglutarate target protein, pendrin.

## Discussion

These studies provide the first examination of NBCe1's role in renal organic anion metabolism. NBCe1 deletion decreased urinary citrate excretion, similar to that expected from the concomitant metabolic acidosis. However, the mechanism of the change in citrate excretion differed from that observed with metabolic acidosis alone, with NBCe1 deletion decreasing and experimental metabolic acidosis increasing proximal tubule apical NaDC‐1 expression. NBCe1 deletion also altered urinary 2‐oxoglutarate excretion, but the change, increased excretion, was the exact opposite of the response to metabolic acidosis alone. The increased 2‐oxoglutarate excretion caused by NBCe1 deletion was not associated with significant abnormalities in the expression of key enzymes and transporters involved in 2‐oxoglutarate metabolism. Finally, the lack of urine alkalinization in NBCe1 deletion mice despite high 2‐oxoglutarate excretion correlates with decreased pendrin expression and appears to be independent of changes in Oxgr1 expression.

The finding that NBCe1 expression is necessary for normal organic anion metabolism expands NBCe1's role in acid‐base homeostasis. NBCe1 is a basolateral Na^+^‐coupled, electrogenic bicarbonate transporter and is the primary mechanism of basolateral bicarbonate transport in the proximal tubule (Aalkjaer et al. 2004; Boron 2006; Kurtz and Zhu 2013; Zhuo and Li 2013). Its inhibition decreases proximal tubule bicarbonate reabsorption (Boron 2006; Zhuo and Li 2013) and its deletion leads to severe proximal renal tubular acidosis (Gawenis et al. 2007). NBCe1 is also necessary for normal renal ammonia metabolism (Handlogten et al. 2015). NBCe1 deletion decreases ammonia excretion, despite the concomitant metabolic acidosis that should stimulate ammonia excretion, and this abnormal ammonia metabolism response involves abnormal proximal tubule phosphate‐dependent glutaminase, phospho*enol*pyruvate carboxykinase, and glutamine synthetase expression (Handlogten et al. 2015).

The current studies examined the effects of NBCe1 deletion on citrate because this organic anion has multiple important roles in mammalian physiology. It is an important component of the Krebs cycle, and its metabolism generates intracellular bicarbonate. Consequently, urinary citrate excretion enables alkali excretion without increasing urine pH (Baruch et al. 1975; Simpson 1983; Hamm and Simon 1987; Cheema‐Dhadli et al. 2002). Moreover, citrate excretion is increased by metabolic alkalosis and decreased by metabolic acidosis (Balagura‐Baruch et al. 1973; Baruch et al. 1975; Hamm and Simon 1987; Lin et al. 1998; Cheema‐Dhadli et al. 2002). Citrate excretion also has an important role in prevention of calcium nephrolithiasis; this is because citrate chelates urinary calcium, decreasing the ionized calcium concentration and inhibiting crystal nucleation and stone growth (Pak 1991; Wasserstein 1998; Pearle 2001; Byer and Khan 2005; Aggarwal et al. 2013).

The current studies show that NBCe1 expression regulates key components of renal citrate metabolism. Citrate is freely filtered by the glomerulus, and the major mechanism regulating citrate excretion is thought to involve proximal tubule citrate reabsorption (Pajor 1999; He et al. 2004b; Unwin et al. 2004). Under normal circumstances, metabolic acidosis decreases urinary citrate excretion by increasing proximal tubule citrate reabsorption (Simpson 1983; Hamm and Simon 1987). This is associated with increases in NaDC‐1 expression (Hamm and Simon 1987; Aruga et al. 2000; Liu et al. 2010), as we found in the current studies showing increased proximal tubule apical NaDC‐1 expression in response to experimental metabolic acidosis. Moreover, the current studies show that NBCe1 expression is necessary for the increase in proximal tubule NaDC‐1 expression in response to metabolic acidosis. Specifically, in the absence of NBCe1, despite the presence of metabolic acidosis (Gawenis et al. 2007; Handlogten et al. 2015), NaDC‐1 expression decreases, rather than increases. Thus, NBCe1 is necessary for the normal regulation of proximal tubule NaDC‐1 expression.

Urinary citrate excretion decreased in mice with NBCe1 deletion despite the decrease in NaDC‐1 expression. This suggests activation of alternative citrate reabsorptive mechanisms and is consistent with the observation of substantial residual renal tubular citrate reabsorption in mice with NaDC‐1 deletion (Teran et al. 2015). This alternative citrate transport mechanism is likely to be related to the recently described calcium‐regulated apical citrate transport activity (Hering‐Smith et al. 2011, 2014), which is known to be stimulated by extracellular acidosis (Hering‐Smith et al. 2000).

Expression of NaDC‐3, another renal citrate transporter, was increased by experimental metabolic acidosis, whereas NBCe1 deletion decreased NaDC‐3 expression. Thus, NBCe1 expression is necessary for normal expression of both NaDC‐1 and NaDC‐3. However, the abnormal NaDC‐3 expression is unlikely to be a major mechanism of the suppressed citrate excretion in NBCe1 KO mice. Specifically, NaDC‐3 is expressed in the basolateral plasma membrane (Wang et al. 2003; Bai et al. 2006) where, because it mediates cellular citrate uptake, it is unlikely to function in luminal citrate reabsorption.

A second major finding of the current studies is that NBCe1 expression is necessary for normal 2‐oxoglutarate metabolism. NBCe1 deletion induced a dramatic increase in 2‐oxoglutarate excretion, which contrasted with the decreased 2‐oxoglutarate excretion that metabolic acidosis induced. Because cellular 2‐oxoglutarate metabolism generates bicarbonate, the increased urinary excretion in NBCe1 KO mice is equivalent to urinary alkali excretion, and may be contributing to the severe metabolic acidosis observed in these mice (Gawenis et al. 2007; Handlogten et al. 2015). Thus, abnormal 2‐oxoglutarate metabolism occurs with NBCe1 deletion, cannot be explained by the concomitant metabolic acidosis, and contributes to the generation of the severe metabolic acidosis.

At present, the mechanism of altered 2‐oxoglutarate excretion, either with NBCe1 deletion or with experimental metabolic acidosis, is unclear. The changes in 2‐oxoglutarate excretion could not be explained by changes in the expression of the primary basolateral 2‐oxoglutarate transporter, NaDC‐3, either the glutaminase I or glutaminase II pathways that lead to 2‐oxoglutarate production, or in the primary enzyme complex involved in renal metabolism of 2‐oxoglutarate to succinate, the oxoglutarate dehydrogenase complex. The organic anion transporter, OAT10, is expressed in the proximal tubule apical membrane (Bahn et al. 2008), and in another model in which 2‐oxoglutarate excretion is increased, SPAK‐kinase deletion, OAT10 expression was increased (Grimm et al. 2015). However, in mice with NBCe1 deletion, there was a slight decrease in OAT10 mRNA expression, which cannot explain the dramatic increase in 2‐oxoglutarate excretion. Thus, changes in the expression of known 2‐oxoglutarate transporters and enzymes involved in 2‐oxoglutarate production do not explain the changes in 2‐oxoglutarate excretion observed with NBCe1 deletion or with experimental metabolic acidosis.

This study also provides new information regarding the role of OAT10 in 2‐oxoglutarate metabolism. OAT10 is expressed in the proximal tubule (Bahn et al. 2008), and changes in OAT10 expression have been proposed to be an important mechanism regulating 2‐oxoglutarate excretion (Grimm et al. 2015). However, in this study experimental metabolic acidosis decreased 2‐oxoglutarate excretion but did not significantly alter OAT10 expression, and NBCe1 deletion dramatically increased 2‐oxoglutarate excretion despite decreased OAT10 expression. These observations suggest that altered OAT10 expression is unlikely to be the primary mechanism regulating 2‐oxoglutarate excretion in these two conditions. However, we cannot exclude the possibility that OAT10 is regulated primarily through posttranslational mechanisms, enabling increased 2‐oxoglutarate excretion despite absence of changes in mRNA expression.

2‐oxoglutarate has been proposed to contribute to acid‐base homeostasis as a paracrine signaling molecule that stimulates distal epithelial cell bicarbonate secretion (Peti‐Peterdi 2013; Tokonami et al. 2013). This mechanism appears to involve activation of the G‐protein coupled receptor, Oxgr1 (He et al. 2004a), and stimulation of pendrin‐mediated bicarbonate secretion (Tokonami et al. 2013). Increased 2‐oxoglutarate excretion resulting from NBCe1 deletion, however, does not appear to be associated with increased collecting duct bicarbonate secretion. In particular, NBCe1 deletion causes urine pH to be more acidic, averaging ~4.2 (Handlogten et al. 2015), a finding inconsistent with 2‐oxoglutarate significantly stimulating distal bicarbonate secretion. This lack of urine alkalinization in NBCe1 KO mice does not appear to result from decreased Oxgr1 expression. Oxgr1 activation has been suggested to stimulate urinary bicarbonate excretion by activating pendrin‐mediated bicarbonate secretion (Peti‐Peterdi 2013; Tokonami et al. 2013), and NBCe1 deletion decreased pendrin expression significantly. This is likely the result of the metabolic acidosis that develops with NBCe1 deletion (Gawenis et al. 2007; Handlogten et al. 2015), as multiple previous studies have shown that metabolic acidosis decreases pendrin expression (Frische et al. 2003; Petrovic et al. 2003; Purkerson et al. 2010). Thus, decreased pendrin expression, rather than altered Oxgr1 expression, likely precludes significant 2‐oxoglutarate‐stimulated bicarbonate secretion in mice with NBCe1 deletion.

The studies in the current manuscript also provide additional insight into the renal response to metabolic acidosis. First, chronic metabolic acidosis decreases 2‐oxoglutarate excretion (67 and current study). Second, we show for the first time that metabolic acidosis decreases Oxgr1 expression. Because 2‐oxoglutarate stimulates HCO_3_^−^ secretion through Oxgr1 activation (Tokonami et al. 2013), decreased Oxgr1 expression is likely to inhibit 2‐oxoglutarate‐dependent stimulation of HCO_3_^−^ secretion. Finally, several previous studies show that metabolic acidosis decreases pendrin expression (Frische et al. 2003; Petrovic et al. 2003; Purkerson et al. 2010). These findings suggest that metabolic acidosis inhibits expression of all three components of this 2‐oxoglutarate‐dependent paracrine signaling pathway, signaling molecule (2‐oxoglutarate), receptor (Oxgr1), and target protein (pendrin).

The NBCe1 knockout mice used in the current studies are a different strain (mixed 129S6/SvEv and Black Swiss) than the adult wild‐type mice (C57Bl/6) used to assess independent effects of metabolic acidosis. However, it is highly unlikely that the strain differences account for the profound differences observed. Changes in citrate excretion in response to metabolic acidosis have been shown in multiple species, including humans, rabbits, cats, rats, and mice (Östberg 1931; Martensson 1940; Crawford et al. 1959; Nissim and Yudkoff 1990; Aruga et al. 2000; Liu et al. 2010), and studies in both rats and mice have shown metabolic acidosis increases NaDC‐1 expression (Aruga et al. 2000; Boehmer et al. 2004; Liu et al. 2010). Similarly, decreased 2‐oxoglutarate excretion in response to metabolic acidosis has been shown in multiple species (Ferrier et al. 1985; Martin et al. 1989; Cheema‐Dhadli et al. 2002; Tokonami et al. 2013). These consistent responses make it unlikely that the dramatically different responses in the NBCe1 knockout mice are due to strain‐dependent differences.

In summary, the current studies demonstrate important new findings regarding the role of NBCe1 in the regulation of multiple aspects of proximal tubule acid‐base physiology. Previous studies have shown critical roles for NBCe1 in proximal tubule bicarbonate reabsorption and ammonia metabolism. The current studies show that NBCe1 expression is also necessary for normal expression of the proximal tubule organic anion transporters, NaDC‐1 and NaDC‐3, and for normal 2‐oxoglutarate metabolism. The abnormal urinary 2‐oxoglutarate excretion does not cause urine alkalinization, as would otherwise be expected, because of downregulation of collecting duct pendrin expression. Thus, NBCe1 expression is necessary for multiple components of the proximal tubule contribution to acid‐base homeostasis, namely bicarbonate reabsorption, ammonia metabolism, and organic anion metabolism.

## Conflict of Interest

None declared.

## Note

1 The NBCe1 pups used for examination of the effect of NBCe1 deletion are not weaned at age of 8 days. As a result, it is not feasible to examine the effect of chronic metabolic acidosis in mice this age.
